# Single cell mutational analysis of *PIK3CA* in circulating tumor cells and metastases in breast cancer reveals heterogeneity, discordance, and mutation persistence in cultured disseminated tumor cells from bone marrow

**DOI:** 10.1186/1471-2407-14-456

**Published:** 2014-06-19

**Authors:** Glenn Deng, Sujatha Krishnakumar, Ashley A Powell, Haiyu Zhang, Michael N Mindrinos, Melinda L Telli, Ronald W Davis, Stefanie S Jeffrey

**Affiliations:** 1 College of Life Science and Chemistry, Wuhan Donghu University, Wuhan, P. R. China; 2 Division of Surgical Oncology, Stanford University School of Medicine, Stanford, CA 94305, USA; 3 Stanford Genome Technology Center, Stanford University School of Medicine, Palo Alto, CA 94304, USA; 4 Division of Medical Oncology, Stanford University School of Medicine, Stanford, CA 94305, USA; 5 Division of Gynecologic Oncology, Stanford University School of Medicine, Stanford, CA 94305, USA; 6 Department of Pathology, Stanford University School of Medicine, Stanford, CA 94305, USA

**Keywords:** Cancer cell culture, Circulating tumor cells (CTCs), Disseminated tumor cells (DTCs), Heterogeneity, Mutation analysis, *PIK3CA*, Single cell analysis

## Abstract

**Background:**

Therapeutic decisions in cancer are generally guided by molecular biomarkers or, for some newer therapeutics, primary tumor genotype. However, because biomarkers or genotypes may change as new metastases emerge, circulating tumor cells (CTCs) from blood are being investigated for a role in guiding real-time drug selection during disease progression, expecting that CTCs will comprehensively represent the full spectrum of genomic changes in metastases. However, information is limited regarding mutational heterogeneity among CTCs and metastases in breast cancer as discerned by single cell analysis. The presence of disseminated tumor cells (DTCs) in bone marrow also carry prognostic significance in breast cancer, but with variability between CTC and DTC detection. Here we analyze a series of single tumor cells, CTCs, and DTCs for *PIK3CA* mutations and report CTC and corresponding metastatic genotypes.

**Methods:**

We used the MagSweeper, an immunomagnetic separation device, to capture live single tumor cells from breast cancer patients’ primary and metastatic tissues, blood, and bone marrow. Single cells were screened for mutations in exons 9 and 20 of the *PIK3CA* gene. Captured DTCs grown in cell culture were also sequenced for *PIK3CA* mutations.

**Results:**

Among 242 individual tumor cells isolated from 17 patients and tested for mutations, 48 mutated tumor cells were identified in three patients. Single cell analyses revealed mutational heterogeneity among CTCs and tumor cells in tissues. In a patient followed serially, there was mutational discordance between CTCs, DTCs, and metastases, and among CTCs isolated at different time points. DTCs from this patient propagated *in vitro* contained a *PIK3CA* mutation, which was maintained despite morphological changes during 21 days of cell culture.

**Conclusions:**

Single cell analysis of CTCs can demonstrate genotypic heterogeneity, changes over time, and discordance from DTCs and distant metastases. We present a cautionary case showing that CTCs from any single blood draw do not always reflect metastatic genotype, and that CTC and DTC analyses may provide independent clinical information. Isolated DTCs remain viable and can be propagated in culture while maintaining their original mutational status, potentially serving as a future resource for investigating new drug therapies.

## Background

Clinical use of some newer or investigational drug therapies in cancer requires that primary tumors be assayed for specific mutations associated with response or lack of response [1-5]. However, not only are primary tumors known to be mutationally heterogeneous [6-8], new mutations may become apparent in recurrent tumors, emerging during disease progression [9-11]. Yet sequential biopsy and evaluation of molecular biomarkers and mutations in metastases is not routinely done, even during clinical trials [11], largely due to the multiplicity and internal location of many metastases (such as liver, lung, and/or brain metastases in breast cancer), and potential morbidity associated with sequential biopsy. An appealing alternative is a “liquid biopsy” with CTC capture and characterization [12,13]. As they are easily accessible by simple blood draw, CTCs can be sequentially sampled at multiple time points during the course of disease for biomarker or genotype determination. Moreover, it is hoped, but not ascertained, that CTCs represent mixtures of tumor cells that reflect the full spectrum of molecular phenotypes and genotypes present in multiple metastases.

Following a slightly different tack, some groups are also investigating biomarker and genetic characteristics of DTCs from bone marrow [14-16], which are postulated to serve as a reservoir for active and dormant tumor cells [12,17]. However, genetic analyses of mutations in CTCs and DTCs are still in an early discovery stage, having been done on few patients, so clinical significance and utility is postulated but remains unproven. Moreover, it is not known whether CTC analysis can replace DTC analysis for there is, as yet, incomplete understanding of the relationship between these two populations [18].

In the present study, we used a previously described magnetic separation technology that isolates live single cells [19-21] for mutation analysis of single cells from different compartments in metastatic breast cancer patients and also demonstrate growth in culture of patient DTCs from bone marrow. For single CTC/DTC/tumor mutation analysis, we have chosen to interrogate exons 9 and 20 of the *PIK3CA* gene, one of the most frequently mutated genes in breast cancer [22-25]. We demonstrate that this mutation can be detected in single tumor cells isolated from breast cancer patient primary tumor, blood, bone marrow, and metastases, and track mutational status of CTCs over time in a metastatic breast cancer case example and in cultured DTCs from this patient. While we have previously shown that individual CTCs in breast cancer, even from the same blood draw, are transcriptionally heterogeneous [21], here we investigate mutational heterogeneity and concordance among CTCs, DTCs, and single tumor cells from primary tumors and metastases. In particular, for CTCs to be ultimately used to guide drug selection, we hypothesized that CTCs should indeed contain the mutational changes found in metastases. However, our results were surprising and we present here a case that provides a cautionary note that CTCs from any one blood draw alone may not always represent the mutational status of tumor cells in bone marrow or distant metastases.

## Methods

### Ethics statement

This study protocol was approved by Stanford’s Human Subjects Research and Institutional Review Board (Protocol 5630). Written informed consent was explained and signed by all participating patients prior to sample collection.

### Tumor cell isolation, staining, and culture

Single cell suspensions used for MagSweeper tumor cell isolation were prepared from primary and metastatic tissue from breast cancer patients. Tumor chunks were finely minced, gently pulled to release single cells or small cell clusters, filtered through a 70 micron mesh followed by centrifugation of the filtrate at 1900 g. The supernatant was discarded and the pellet was resuspended in 1x trypsin (Invitrogen/Life Technologies, Carlsbad, CA, USA) for 5–10 minutes. DMEM culture media with 10% FBS (Gibco/Life Technologies, Carlsbad, CA, USA) was added to stop the trypsin reaction. The target single tumor cells were labeled with EpCAM-conjugated microbeads and isolated by the MagSweeper as previously described [19,21]. Individual tumor cells were aspirated under direct microscopic visualization (Axio Observer A1, Zeiss, Thornwood, NY, USA). Authenticated MCF7 and BT474 human breast cancer cell lines (ATCC, Manassas, VA, USA) were grown in DMEM and trypsinized to release single cells, which were then isolated by the MagSweeper and manually aspirated as single cells.

For immunostaining assays, EpCAM-captured cells were treated with DNase I Solution (StemCell Technologies, Vancouver, BC, Canada) to remove the DNA-linker on the magnetic microbeads, and placed on slides. Tumor cells were defined by immunostain assay [26-28] as cells that stained positive for purified anti-cytokeratin (CK+) CAM 5.2 (BD Biosciences, San Jose, CA, USA) and DAPI nuclear stain (DAPI+) (VECTASHIELD Mounting Medium with DAPI, Vector Laboratories, Burlingame, CA, USA), and negative for CD45 (CD45-) (CD45 Ab-1/Bra55, NeoMarkers Lab Vision, Thermo Fisher Scientific, Kalamazoo, MI, USA). White blood cells (WBCs) from patients were collected using the MagSweeper and CD45 Dynabeads (Invitrogen, Carlsbad, CA, USA) and similarly immunostained.

Bone marrow aspirates obtained from the clinic were filtered through a 70 micron mesh to eliminate debris; the passed through solution was adjusted to 10 ml by adding DMEM. DTCs from the prepared bone marrow sample solution were isolated by the MagSweeper and hundreds of DTCs were directly cultured in DMEM with 10% FBS and 50 μg/ml of penicillin-streptomycin (Gibco/Life Technologies, Carlsbad, CA, USA). The cells were then identified by immunostaining assays. The DTCs were cultured for 21 days and single cells were assayed for mutation detection.

Blood sample collection and MagSweeper isolation of CTCs were isolated as previously described [19,21]. White blood cells (n = 15, from the blood of Patient 12) were collected using anti-CD45 microbeads.

### Mutation analysis

Single tumor cells from 17 breast cancer patients were lysed by incubation with a 1:10 dilution of proteinase K (Qiagen, Valencia, CA, USA) in individual PCR tubes for 20 min at 65°C in 10 μl of 1x GeneAmpPCR buffer II (Applied Biosystems/Life Technologies, Carlsbad, CA, USA). In one of the 17 patients, additional tumor cell clusters were tested from slides sectioned from formalin-fixed paraffin-embedded (FFPE) blocks of primary tumor and a lung metastasis with location confirmed by hematoxylin and eosin (H&E) stain. For tumor tissue fixed on slides, diluted proteinase K was placed on the targeted tissue section area of the slide. The solution was heated at 65°C for 20 min and collected for DNA mutation analysis. The DNA from single CTC, DTC, or tumor tissue cells was then pre-amplified for exons 9 and 20 of the *PIK3CA* gene, using Pfu Ultra DNA polymerase (Agilent Technologies, Santa Clara, CA, USA) with one set of primers for each exon (exon 9 forward primer: CTGTGAATCCAGAGGGGA, reverse primer: CAGAGAATCTCCATTTTAGCAC; exon 20 forward primer: GGAATGCCAGAACTACAATCTTTTG, reverse primer: CCTATGCAATCGGTCTTTGC). The reaction was amplified for 30 cycles at 94°C, 55°C, and 72°C for 30 sec per cycle for each temperature. The pre-amplified products were then diluted 1:10 in distilled water (DW) and 5 μl of the diluted products were used for a single 50 μl PCR using the same primers as above for exon 9, and an internal primer pair for exon 20 that decreased non-specific background amplification (forward primer: GTGGAATCCAGAGTGAGC, reverse primer: TTGCATACATTCGAAAGACC) [25]. PCR products were checked by 2% agarose gel against a GeneRuler 50 bp DNA Ladder (Frementas, Glen Burnie, MD, USA) and sequenced by BigDye Terminator v3.1 Cycle Sequencing Kits according to the manufacturer’s recommended protocol (Applied Biosystems/Life Technologies, Carlsbad, CA, USA). The sequenced results were analyzed with Sequencher 4.8 software (Gene Codes Corporation, Ann Arbor, MI, USA). DW was used as a negative template control. Single authenticated MCF7 and BT474 cells were used as amplification and sequencing controls: MCF7 cells contain a G1633A mutation in exon 9 and are wild type in exon 20; BT474 cells contain an exon 1 mutation but are wild type in exons 9 and 20. BT20 cells, which contain the A3140G mutation in exon 20, served as a positive control for sequencing of exon 20 [29]. Fifteen single WBCs isolated from blood samples also served as wild type control for sequencing.

## Results

### Patient samples and tumor cell identification

**Figure 1:**
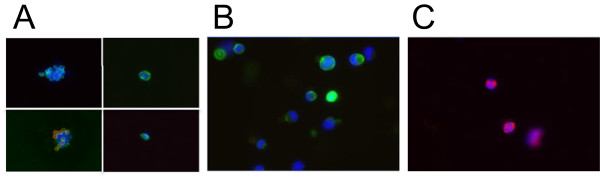
**Single cell isolation and identification.** Individual tumor cells (TCs) from primary or metastatic tissue, blood, or bone marrow were isolated by the MagSweeper and immunostained (200×). Panel **(A)** shows examples of CTCs and DTCs (CK+, CD45-, DAPI+); small round circles in two frames of **(A)** are autofluorescing residual magnetic microbeads; Panel **(B)** shows an example of TCs from tumor tissue (CK+, CD45-, DAPI+); Panel **(C)** shows examples of WBCs (CK-/very weak, CD45+, DAPI+). Green = cytokeratin; red = CD45; blue = DAPI nuclear stain.

From a group of 17 breast cancer patients, single tumor cells were collected using anti-EpCAM microbeads and the MagSweeper from 30 blood samples, a bone marrow biopsy, and six fresh tumor tissues (Table 1, and Table 2). CTCs from blood, DTCs from bone marrow, and tumor cells from fresh primary and metastatic tumors were defined by immunostain assay as cells that were CK+, CD45-, DAPI+. WBCs collected from the blood of one patient (Patient 12) using anti-CD45 microbeads for use as an additional wild type control for sequencing were confirmed by immunostaining to be CK negative/very weak, CD45 positive, and DAPI positive (Figure 1). Overall, 769 individual CTCs from blood, 75 DTCs from bone marrow, and 60 single TCs from fresh primary tumor tissue (chunk and core needle biopsy), lymph node metastasis, and a bone metastasis to the spine were collected. Of these, 185 CTCs, 24 DTCs, 33 single TCs from fresh primary and metastatic tissues (total 242 single cells) were collected for mutational analysis. Additional tumor cell clusters from two FFPE samples of primary tumor and a lung metastasis were also analyzed for *PIK3CA* mutations (Table 3).

**Table 1:** Single cell mutation analysis of CTCs

**Patient ID**	**Sample ID**	**Sample type**	Number CTCs analyzed/total CTCs collected*	**Number CTCs with *PIK3CA *** **exon 9 mutation**	**Number CTCs with *PIK3CA *** **exon 20 mutation**
1	1-1	Blood	3/13	0	0
1	1-2	Blood	3/9	0	0
1	1-3	Blood	4/9	0	0
2	2-1	Blood	3/20	0	0
2	2-2	Blood	2/6	0	0
2	2-3	Blood	5/50	0	0
3	3-1	Blood	3/14	0	0
4	4-1	Blood	2/8	0	0
5	5-1	Blood	2/3	0	0
6	6-1	Blood	8/75	0	0
7	7-1	Blood	5/100	0	0
8	8-1	Blood	5/60	0	0
9	9-1	Blood	2/7	0	0
10	10-1	Blood	5/200	0	0
11	11-1	Blood	5/50	0	0
12	12-1	Blood	3/6	0	0
12	12-2	Blood	2/2	0	0
12	12-3	Blood	4/4	0	0
12	12-4	Blood	13/13	0	0
12	12-5	Blood	20/20	10	0
12	12-6	Blood	7/9	2	0
12	12-7	Blood	27/27	0	ND
12	12-8	Blood	50/50	0	ND
12	12-9	Blood	0/0	-	-
12	12-10	Blood	2/2	0	0
13	13-1 (BL)	Blood	0/0	-	-
14	14-1 (BL)	Blood	0/6	-	-
15	15-1 (BL)	Blood	0/6	-	-
16	16-1 (BL)	Blood	0/0	-	-
17	17-1 (BL)	Blood	0/0	-	-
TOTALS					
17 patients	30 samples	-	185/769	12	0

*CTCs* circulating tumor cells, *ND* not determined.

*CTCs not analyzed for mutations were used for transcriptional analysis [21], immunostaining, or other molecular assays.

**Table 2:** Single cell mutation analysis of DTCs or primary or metastatic tumor cells

**Patient ID**	**Sample ID**	**Sample type**	Number DTCs analyzed/total DTCs collected*	Number TCs analyzed/total TCs collected*	**Number DTCs or TCs with *PIK3CA *** **exon 9 mutation**	**Number DTCs or TCs with *PIK3CA *** **exon 20 mutation**
12	12-11 (T)	L2 bone metastasis (spine)	-	8/10	8	0
12	12-12 (T)	Bone marrow	24/75	-	24	0
13	13-1 (T)	Primary tumor	-	5/10	0	0
14	14-1 (T)	Primary tumor (core bx)	-	5/10	1	0
15	15-1 (T)	LN metastasis	-	5/10	3	0
16	16-1 (T)	Primary tumor	-	5/10	0	0
17	17-1 (T)	Primary tumor	-	5/10	0	0
TOTALS						
6 patients	7 samples	-	24/75	33/60	36	0

*DTCs* disseminated tumor cells from bone marrow, *TCs* tumor cells from primary or metastatic tumors;

*LN* lymph node, *L2* second lumbar vertebra, *Bx* biopsy, *ND* not determined.

*DTCs or TCs not analyzed for mutations were used for immunostaining or other molecular assays.

**Table 3:** Mutation analysis from formalin-fixed paraffin-embedded (FFPE) tissue

**Patient ID**	**Sample type**	***PIK3CA***	***PIK3CA***
		**Exon 9**	**Exon 20**
12	Primary tumor	Wild type	Wild type
12	Lung metastasis	Mutation present	Wild type

### DTC culture

**Figure 2:**
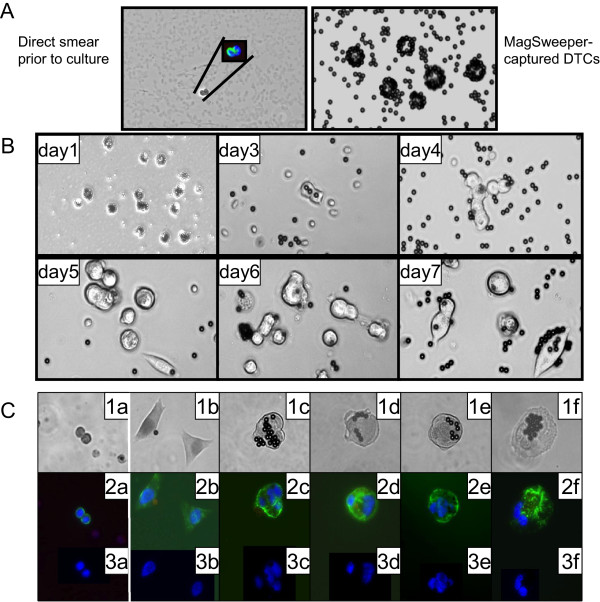
**Live DTCs captured by the MagSweeper and propagated *in vitro. *(A)** DTCs detected in fresh smear of bone marrow aspirate among a background of red blood cells (RBCs) and white blood cells (WBCs) (left panel); unstained EpCAM-captured DTCs from bone marrow aspirate (right panel); small black circles are EpCAM-conjugated microbeads. **(B)** EpCAM-captured tumor cells (200×) grown in culture for three weeks: tumor cells proliferated days 1–4, but began changing shape on day 5. **(C)** Tumor cells identified by immunostaining at different timepoints during cell culture period (a = day 1; b-f = day 21 at 200×); upper panel 1 shows cell morphology by brightfield; middle panel 2 shows immunostain images (all cells were CK+, CD45- and DAPI+); lower panel 3 shows varying nuclear morphology of cells between day 1 day 21. Green = cytokeratin, red = CD45, blue = DAPI nuclear stain.

The MagSweeper facilitates the isolation of viable target cells that can be grown in culture. Pooled bead-captured DTCs (Figure 2A) showed distinct morphological changes when grown over a 21-day cell culture period (Figure 2B and C). At the time of bone marrow biopsy, DTCs had round shapes when examined fresh and after capture by the MagSweeper (Figure 2A). However, onward from day 5, cell shapes started varying, with some changing to elongated or irregular shapes, although immunostaining assays at different stages demonstrated that all cultured cells remained CK+, CD45- and DAPI+, as expected of tumor cells (Figure 2B and C).

### *PIK3CA* gene mutations

**Figure 3:**
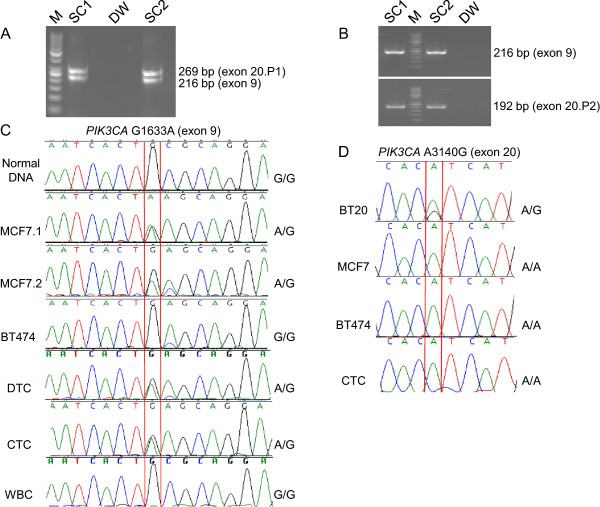
**Single cell *PIK3CA *mutation detection. (A)** Target DNA from two single tumor cells (SC1, SC2) successfully pre-amplified by PCR with the expected bands for *PIK3CA* exon 9 (216 bp) and exon 20 (269 bp). **(B)** Second round of amplification using the pre-amplified PCR products from **(A)** as new DNA templates to separately amplify exon 9 (using original primers, 216 bp) and exon 20 (using internal primers, 192 bp). P1 = PCR product from first round of amplification; P2 = PCR product from second round of amplification. **(C)** Sanger sequencing results for *PIK3CA* mutation G1633A on exon 9: two MCF7 single cells shown here carry the G1633A heterozygous mutation; Normal DNA, BT474 single cells, and single WBCs are wild type (G/G); *PIK3CA* G1633A mutations were detected in single DTCs and CTCs from breast cancer patient 12. The G1633A mutation was distinguishable in the chromatogram from the adjacent A1634C peak from a known pseudogene on chromosome 22 that may be co-amplified with these primers [34], as in sample MCF7.2. **(D)** Sanger sequencing results for PIK3CA mutation A3140G on exon 20: BT20 cells show the mutation but MCF7 cells, BT474 cells, and CTCs are wildtype for this mutation hotspot.

To first assess our mutation detection method, targeted DNA from single tumor cells or cells from cell lines was successfully pre-amplified by PCR and the expected bands were confirmed for *PIK3CA* exons 9 (216 bp) and 20 (269 bp) (Figure 3A). Using the pre-amplified PCR products as new DNA templates, exons 9 and 20 were separately amplified for sequencing, amplifying exon 9 using the original primers and exon 20 with internal primers. Single PCR bands for each exon verified the specificity of the amplification prior to sequencing (Figure 3B). A known heterozygous *PIK3CA* exon 9 mutation, G1633A (E545K), was identified in replicate samples of single MCF7 cells (positive control), single DTCs, and single CTCs, but was not detected in single WBCs (wild type/negative control) or single BT474 cells (which carry a *PIK3CA* mutation in exon 1, but no mutations in exons 9 or 20, another negative control) [30-33] (Figure 3C). Note that A1634C (E545A) in the chromatogram in Figure 3B sometimes showed a pseudogene that may be co-amplified with these primers [34]. No exon 20 mutation was identified in negative control MCF7 and BT474 cells, any patient tumor cells, or captured WBCs among our samples, although sequencing of BT20 cells (positive control) detected the expected exon 20 mutation at A3140G (H1047R), confirming the accuracy of our sequencing method (Figure 3D).

*PIK3CA* mutation analysis was then performed on the 242 EpCAM-captured single tumor cells (185 CTCs, 24 DTCs, and 33 tumor cells). Three out of 17 (18%) patients showed the *PIK3CA* exon 9 G1633A mutation in tissue; no exon 20 mutations were detected (Table 1, Table 2 and Table 3). Of these three patients (Patient 12, Patient 14, and Patient 15), 33 individual tumor cells obtained from six fresh clinical samples were sequenced and three showed the exon 9 mutation: 8/8 (100%) single tumor cells from a spinal bone metastasis (Patient 12); 1/5 (20%) single tumor cells from a primary tumor core biopsy (Patient 14); and 3/5 (60%) single tumor cells from a metastatic lymph node (Patient 15). Not unexpectedly, and similar to this patient’s bone metastasis, all 24 (100%) sequenced single DTCs collected from a 3 ml aspirate of Patient 12’s tumor-replaced bone marrow contained the exon 9 mutation (the bone marrow aspirate had been done to determine if tumor overgrowth was causing her pancytopenia, and the DTCs detected were too numerous to count). Interestingly, the FFPE sample of Patient 12’s primary tumor was wild type, but a tissue section of her lung metastasis also showed the *PIK3CA* exon 9 mutation.

**Figure 4:**
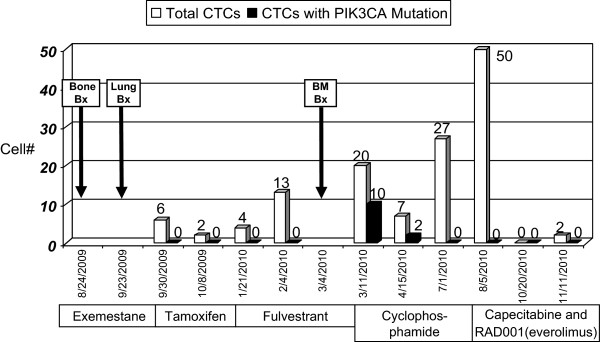
***PIK3CA *G1633A mutation detection in CTCs and tissues over time.** One patient, Patient 12, with progressive metastatic breast cancer underwent tissue evaluation and *PIK3CA* sequencing of a bone (spine) metastasis, lung metastasis, bone marrow biopsy and aspirate (DTCs), and sequential blood draws for one and a half years. Eight out of 10 EpCAM-captured single cells (two cells did not get PCR products for sequencing) in the core needle biopsy of a bone metastases (Bone Bx) from the lumbar spine carried the *PIK3CA* exon 9 heterozygous mutation G1633A; a lung biopsy (Lung Bx) also showed this mutation; 24 out of 24 EpCAM-captured single cells analyzed from the bone marrow biopsy (BM Bx) carried the heterozygous mutation (EpCAM-captured DTCs retrieved from the bone marrow aspirate). The G1633A mutation was detected only twice in CTCs captured from nine blood samples: one week after bone marrow biopsy, with 10/20 EpCAM-captured cells having the mutation, and six weeks after bone marrow biopsy, with 2/7 EpCAM captured cells having the mutation. Drug treatments are noted (RAD001 = everolimus, an mTOR inhibitor). Note that increasing CTC counts dramatically decreased after treatment with capecitabine and everolimus.

CTCs from Patient 12 were captured periodically over 14 months, during changing treatment of her metastatic breast cancer (Table 1, Table 2, Table 3 and Table 4, Figure 4). Remarkably, in this patient with mutant DTCs in her bone marrow and mutant tumor cells in her spinal bone metastasis and lung metastasis, CTCs in some blood draw samples were discordant and did not show the expected mutation. Notably, of ten blood samples collected from this patient over time, only nine blood samples contained capturable CTCs; of the 128 CTCs sequenced from these nine samples, mutant CTCs were detected in only two blood draws: at one and six weeks after bone marrow biopsy, with mutant cells comprising 50% (10/20) and 29% (2/7) of EpCAM-captured cells, respectively (Table 1, Table 2, Table 3 and Table 4, Figure 4). As expected, all 15 WBCs analyzed from this patient were wild type. Our single cell data thus indicate that not only may there be mutational heterogeneity within a sample, there may be genotypic discordance between CTCs, DTCs and metastases, or between CTC samples isolated at different time points.

**Table 4:** Treatments, sampling times, and biomarkers of tumor cells in different tissue compartments from patient 12 during disease progression

**Date sampled**	**Treatment**	**Tissue compartment**	***ER***	***PR***	***HER2***	***PIK3CA *** **mutation in CTCs or DTCs or TCs (primary tumor or distant metastasis)**
09/2002	N/A	Primary Tumor	1+ rare	2+, 10%	Neg (IHC 0)	Wild type^#^
8/24/2009	Exemestane	L2 vertebra bone metastasis (spine)	Negative	Insufficient material	Insufficient material	8/8
9/23/2009	Exemestane	Lung metastasis	3+, 95%	Negative	Neg (FISH ratio 0.56)	Mutation present^†^
9/30/2009	Tamoxifen started	Blood	-	-	-	0/3
10/8/2009	Tamoxifen	Blood	-	-	-	0/2
1/21/2010	Fulvestrant	Blood	-	-	-	0/4
2/4/2010	Fulvestrant	Blood	-	-	-	0/13
3/4/2010	Fulvestrant	Bone marrow biopsy	Negative	Negative	Negative	24/24
3/11/2010	Cyclophos-phamide started	Blood	-	-	-	10/20
4/15/2010	Cyclophos-phamide	Blood	-	-	-	2/7
7/1/2010	Cyclophos-phamide	Blood	-	-	-	0/27
8/5/2010	Capecitabine + RAD001 (everolimus)	Blood	-	-	-	0/50
10/20/2010	Capecitabine + RAD001 (everolimus)	Blood	-	-	-	0/0
11/11/2010	Capecitabine + RAD001 (everolimus)	Blood	-	-	-	0/2

*CTCs* circulating tumor cells (from blood), *DTCs* disseminated tumor cells (from bone marrow), *TCs* tumor cells from primary tumor or metastatic site.

All detected *PIK3CA* mutations were heterozygous on exon 9 G1633A (E545K).

^#^Tumor cells obtained and tested from both H&E slide and formalin fixed paraffin-embedded primary tumor tissue.

^†^Tumor cells obtained and tested from H&E slide.

In addition to mutational heterogeneity and discordance, this patient’s clinical pathology showed discordance between different sites when tested for the standard breast cancer biomarkers estrogen receptor (ER), progesterone receptor (PR), and HER2 growth factor receptor: primary tumor and lung were hormone receptor (ER and/or PR) positive, whereas bone marrow and bone were hormone receptor negative (Table 4). No tissue sample was HER2 positive.

Finally, when Patient 12’s DTCs were propagated *in vitro*, all cultured cells maintained the original *PIK3CA* exon 9 mutation when again tested on day 21, despite the observed morphological changes described above.

## Discussion

Investigations are underway exploring the clinical utility of CTCs and DTCs in monitoring cancer patients undergoing systemic drug therapy [35]. However, a recent prospective randomized phase III clinical trial of patients with metastatic breast cancer (SWOG S0500) showed that early change in therapy based on persistently elevated CTC counts three weeks after starting a drug did not change patient outcome - likely due to poor efficacy of the drugs these metastatic patients received after switching therapy [36]. One upshot of this study is the expectation that, in the future, the measurement of CTC biomarkers or genotype, rather than only CTC enumeration, should offer better prediction of which drugs will be efficacious.

However, as demonstrated here, primary tumors and metastases can be heterogeneous, and different metastases do not always display the same biological markers. It is not clinically feasible to biopsy all metastases in a given patient at any one time point, and certainly not for serial sampling over the course of disease, so it is hoped that sampling CTCs will reflect the spectrum of tumor cells requiring treatment in metastatic disease. Here in a patient with progressive metastatic breast cancer, we compared the *PIK3CA* mutation status of sequentially sampled CTCs to that of tumor cells from two biopsied metastases and DTCs from bone marrow. Unfortunately, and surprisingly, our data did not support the premise that CTCs in most blood draws were reflective of metastatic genotype. While we did show that different metastases contained discordant biomarkers (Table 4), *PIK3CA* mutations in CTCs were heterogeneously present in only 2/9 serial blood draws in this patient with multiple distant metastases, two of which (lung and spine) contained tumor cells carrying mutations and whose bone marrow was full of mutant DTCs.

This finding causes pause and suggests that different factors may be at work. One may be that the CTCs analyzed here were captured using the EpCAM cell surface marker. It is postulated that among tumor cells shed from a tissue, many undergo epithelial-mesenchymal transition (EMT), with expression of EMT-associated genes and proteins, and we and others have demonstrated EMT gene and protein expression in CTCs [21,37-39]. Although CTCs in most EMT studies have been captured with EpCAM antibodies, as EMT progresses, EpCAM expression likely diminishes. Thus, CTCs may consist of populations of EpCAM-expressing and non-EpCAM expressing CTCs. One of the limitations of this study is that because of the technology applied, we studied only EpCAM-expressing CTCs. There may be other non-EpCAM-expressing CTCs present in the blood samples that may have shown a mutant genotype not identified in some of the EpCAM-expressing CTCs. While the mutant tumor cells from metastases in our study were also captured using anti-EpCAM magnetic beads, these cells may potentially have been seeded from EpCAM-negative CTCs that had undergone mesenchymal-epithelial-transition after lodging and growing in the metastatic site, with re-expression of EpCAM on their cell surface. We are now testing different cell surface markers and label-free capture technologies to address this issue, which is particularly important because of recent data suggesting an association between EpCAM-negative CTCs and brain metastases [40].

A second limitation or explanation of our findings is that we do not know the role of drug treatment in suppressing the appearance of mutant tumor cells in the circulation. For example, at the time that 50 of Patient 12’s CTCs showed no mutation, the patient was receiving RAD001 (everolimus), an mTOR inhibitor that may be more active against cells carrying *PIK3CA* mutations [41]; her CTC count subsequently dropped to zero, perhaps showing response to therapy over time.

A third limitation may be that sequencing only two common hotspots on the *PIK3CA* gene may miss other genetic variations that could occur during the evolution of progressive metastatic disease, and which may have been shared by the CTCs and metastases. Very recent and exciting work is underway to develop rigorous methods for investigating single cell whole-exome sequencing of CTCs (also captured using MagSweeper technology) [42].

However, given current technology development, our study is important in that it adds a note of caution to using CTCs from only a single blood draw to depict the mutational status in any given patient with metastatic disease for treatment purposes. Treatment decisions based on CTC mutational status should only be done under the auspices of a clinical trial.

While CellSearch™ is the only FDA-approved test for enumeration of CTCs, other CTC capture and characterization technologies, including single cell analysis, are rapidly advancing [43-46]. As sequencing technologies progress and cost decreases, it is anticipated that CTC and DTC genotyping will become more clinically feasible. Genotyping of CTCs or DTCs has generally been performed on pooled samples [47-53]. Studying potentially mixed subpopulations, mutant and wild type, may not be as informative regarding which cells respond to which drugs. However, there are reports describing single cell copy number alterations or mutations in CTCs or DTCs from breast [54-56] or other cancers [12,57-60] using array comparative genomic hybridization and/or sequencing. Like ours, these studies tend to be small, describing only a few cases with aberrant CTC or DTC DNA, but results are encouraging. Tumor cell genotyping at the single cell level may become important clinically because different tumor cell genotypes may be responsive or resistant to different treatments. Thus, capturing and analyzing single tumor cells using deep sequencing of cancer-related genes may lead to even better clarification for selective drug targeting.

However, the lack of mutational discordance between some of the CTC blood draws in Patient 12 and the known presence of mutations in several metastases, raises questions regarding what tumor cells from metastases are released into the circulation, how they may be preferentially impacted by systemic chemotherapy, and whether bone marrow biopsy would be valuable to augment CTC information prior to switching chemotherapy. In the long run, a prospective clinical trial would be needed to determine whether CTCs alone can optimally guide drug therapy and impact survival, or whether assaying both CTCs and DTCs may be better, or whether this is moot until drugs are developed that will ablate metastatic cancer cells.

On a more positive note, we were able to isolate live DTCs and propagate them in culture. Although cell morphology changed over time, mutation status did not. Reports of culturing DTCs are rare [16,61], and this is the second to report *in vitro* conservation of mutational status [16]. As extent of growth of DTCs in culture has been previously associated with poor clinical outcome [16], this lays the groundwork for future *in vitro* drug testing experiments using novel therapeutics against DTCs isolated in treatment-refractory patients.

## Conclusions

Single cell analysis of CTCs can reveal genotypic heterogeneity that may change over time, and can show mutational discordance with DTCs and distant metastases. We present a case suggesting that CTCs may not always reflect the full spectrum of mutations in metastatic disease - or that mutant cells in the circulation may have been more susceptible to the systemic therapy being administered. We postulate that bone marrow may be a more privileged and perhaps chemo-refractory site than the bloodstream, so analyzing both CTCs and DTCs may provide independent clinical information potentially relevant to treatment decisions. Isolated DTCs remain viable and can be propagated in culture while maintaining their original mutational status, and thus may serve as a resource for investigating new drug therapies in the future.

## Abbreviations

CTCs: Circulating tumor cells; DAPI: 4',6-diamidino-2-phenylindole; DTCs: Disseminated tumor cells; EDTA: Ethylenediaminetetraacetic acid; EMT: Epithelial-mesenchymal transition; EpCAM: Epithelial cell adhesion molecule; ER: Estrogen receptor; H&E: Hematoxylin and eosin; HER2: Human epidermal growth factor receptor 2; mTOR: Mammalian target of rapamycin; PCR: Polymerase chain reaction; PIK3CA: Phosphatidylinositol-4,5-bisphosphate 3-kinase, catalytic subunit alpha gene; PR: Progesterone receptor.

## Competing interests

Drs. Stefanie Jeffrey, Ashley Powell, Michael Mindrinos, and Ronald Davis are co-inventors of the MagSweeper device used for tumor cell isolation in this study. Dr Jeffrey has donated her royalties to a non-profit institution. The authors declare that they have no competing interests.

## Authors’ contributions

GD, SK, MM, RD, and SJ conceived of the study and participated in its design and coordination. MT contributed patient samples. GD, AP and HZ isolated CTCs, and GD isolated single tumor cells from all other tissues. GD performed the cell culture experiments and immunostains. GD, SK, and MM performed and analyzed the sequencing assays. GD, SK, MM, and SJ drafted the manuscript. All authors have read and approved the final manuscript.

## Pre-publication history

The pre-publication history for this paper can be accessed here:

http://www.biomedcentral.com/1471-2407/14/456/prepub
